# Validation of a national genetic evaluation for methane emission in Holstein cattle

**DOI:** 10.3168/jdsc.2026-1010

**Published:** 2026-05-15

**Authors:** Saranya G. Narayana, Francesca Malchiodi, Allison Fleming, Colin Lynch, Flavio S. Schenkel, Christine F. Baes, Filippo Miglior

**Affiliations:** 1 Lactanet Canada, Guelph, ON, Canada N1K 1E5; 2 Semex, Guelph, ON, Canada N1H 6J2; 3 Centre for Genetic Improvement of Livestock, University of Guelph, Guelph, ON, Canada N1G 2W1

## Abstract

Lactanet Canada launched a genomic evaluation for methane efficiency for Holsteins in April 2023, utilizing milk mid-infrared–predicted methane (CH_4_) emissions (CH_4MIR_) as a proxy. This study validated the methane efficiency genomic evaluation using genotyped cows with CH_4MIR_ and CH_4_ records from GreenFeed systems (CH_4GF_), along with relative breeding values (RBV) for methane production and methane efficiency from the April 2023 evaluation. In Lactanet's methane efficiency evaluation, a higher RBV indicates more desirable, lower-emitting animals. For the validation, RBV were categorized into quintiles for the CH_4MIR_ dataset and tertiles for the CH_4GF_ dataset to evaluate trends across the RBV distribution. Mean CH_4MIR_ decreased progressively across RBV quintiles for both traits, with all pairwise comparisons among quintiles significantly different. Similarly, CH_4GF_ emissions declined across RBV tertiles, with significant differences observed between the lowest and highest tertiles. Additional analyses using RBV threshold categories confirmed that cows with the highest RBV consistently exhibited lower methane emissions. Linear regression analyses further demonstrated a negative relationship between RBV and methane emissions, supporting the predictive ability of the genomic evaluation. These findings confirm that Canada's genomic evaluation for methane efficiency effectively differentiates cows by methane emission potential, reinforcing its potential as a tool for genetic selection to reduce methane emissions in dairy cattle.

## Highlights

• Validation of Canadian methane efficiency genomic evaluation in Holstein cattle.
• Higher relative breeding values are associated with lower methane emissions.
• Findings support genetic selection to reduce methane emissions in dairy cattle.

## Graphical Abstract

**Figure:**
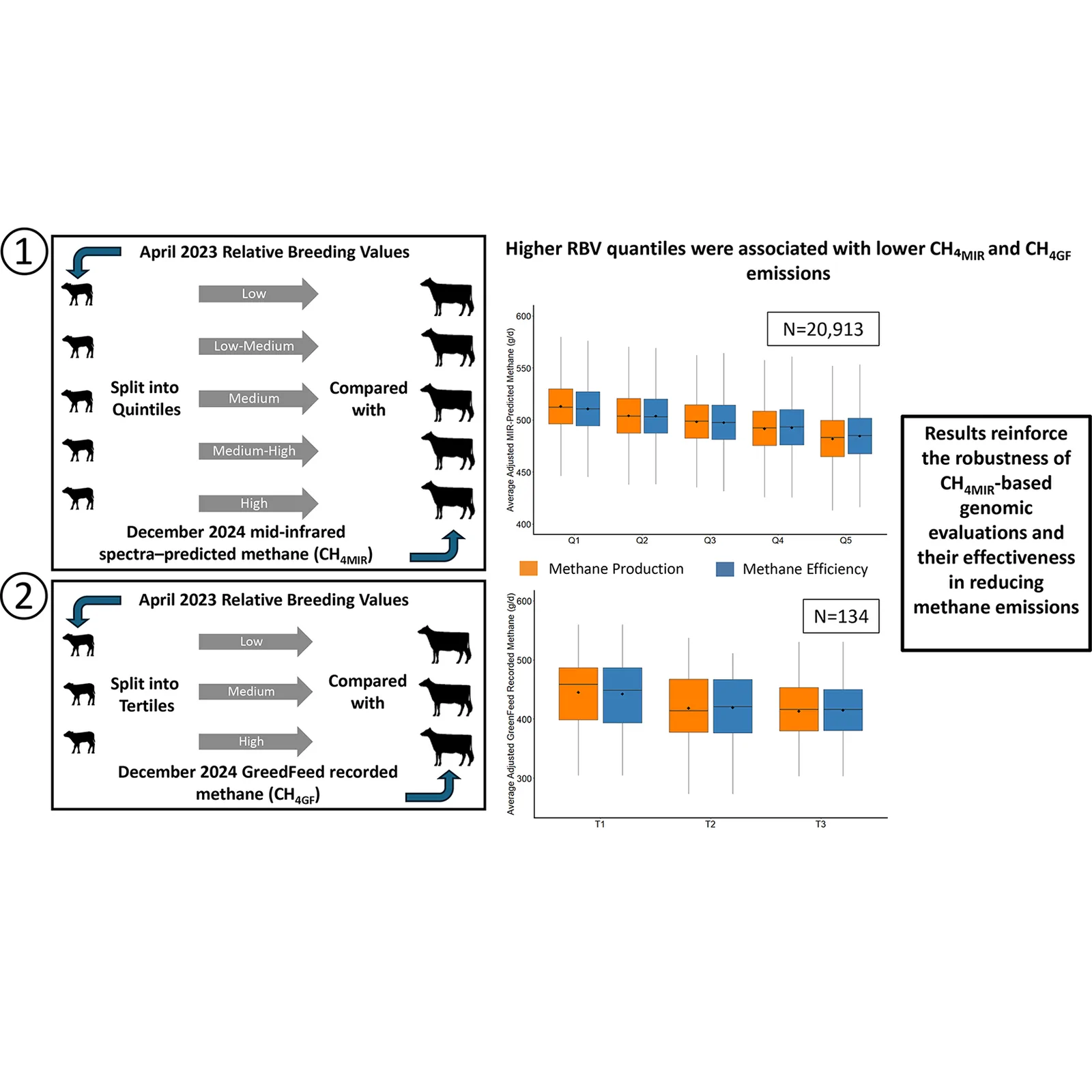
**Summary:** Reducing enteric methane emissions from livestock represents a significant sustainability opportunity, and genetic selection offers an effective strategy to achieve this reduction. Lactanet Canada introduced its methane efficiency (MEF) evaluation in 2023 using milk mid-infrared-predicted methane as a key trait. Here, the MEF evaluation was validated using phenotypic mid-infrared-predicted methane data and methane emissions recorded by GreenFeed systems. Relative breeding values (RBV) were grouped into quintiles for the mid-infrared-predicted methane dataset and into tertiles for the GreenFeed dataset to evaluate trends across the RBV distribution. Mean methane emissions decreased progressively across RBV quintiles for MEF, whereas GreenFeed methane emissions declined across RBV tertiles, with significant differences between the lowest and highest groups. Additional analyses using RBV threshold categories and linear regression supported a negative relationship between RBV and methane emissions. Results confirm that selecting cows with higher RBV for MEF offers a promising long-term strategy for methane mitigation.

The impact of greenhouse gases on climate change has become increasingly concerning. Methane (CH_4_) has a global warming potential 80 times greater than CO_2_ over a 20-year period (IPCC, 2023). In Canada, agriculture accounted for 38% of the country's CH_4_ emissions based on emission inventories (Fouli et al., 2021). The primary source of these emissions is enteric fermentation (33.1%), with a smaller contribution from manure management (4.9%; Fouli et al., 2021; IPCC, 2023). Approximately 95% of CH_4_ is released through eructation and expiration, with the remainder expelled via flatus (Murray et al., 1976; Muñoz et al., 2012). On average, Holstein cows produce 307 to 491 g/d (Denninger et al., 2019; Manzanilla-Pech et al., 2022; Kamalanathan et al., 2023; Rojas de Oliveira et al., 2024) of enteric CH_4_. Hence, global sustainability efforts have placed a strong emphasis on reducing CH_4_ emissions coming from livestock.

Several methods have been developed to quantify CH_4_ emissions, including the GreenFeed system (C-Lock Inc., Rapid City, SD), sniffers, prediction models, respiration calorimetry, and the sulfur hexafluoride (SF_6_) tracer technique (Muñoz et al., 2012; Kebreab et al., 2015; Gunter and Beck, 2018; Manzanilla-Pech et al., 2022; Kamalanathan et al., 2023). Each method has its advantages and limitations, with data collection costs and scalability being common challenges (Hristov et al., 2018; Dressler et al., 2024). As an alternative, mid-infrared (**MIR**) spectroscopy is emerging as a cost-effective and scalable method for estimating CH_4_ emissions in dairy cattle from milk, with a satisfactory level of accuracy (Shadpour et al., 2022; Fresco et al., 2024; McParland et al., 2024; Rojas de Oliveira et al., 2024). Various strategies, including feed additives, microbiome manipulation, and chemical interventions, have been explored to reduce CH_4_ emissions, (Króliczewska et al., 2023; Beauchemin et al., 2025), but genetic selection offers a sustainable solution, as genetic gain is cumulative and permanent. Studies from multiple countries have reported moderate heritability estimates for CH_4_ production (g/d), ranging from 0.11 to 0.45 (Pszczola et al., 2017; Breider et al., 2019; van Breukelen et al., 2023; Rojas de Oliveira et al., 2024), with a meta-analysis of 21 studies estimating a heritability of 0.21 (Ghavi Hossein-Zadeh, 2022).

Lactanet Canada (Guelph, ON) introduced a routine genomic evaluation for methane efficiency (**MEF**) for Holsteins in April 2023, using MIR-predicted CH_4_ production (**CH_4MIR_**; g/d; Van Doormaal et al., 2023; Rojas de Oliveira et al., 2024). The MEF evaluation uses a multiple-trait single-step approach, whereby MEF represents the GEBV for CH_4MIR_ (methane production; **MPR**) adjusted to be genetically independent of milk, fat, and protein yields using a recursive approach (Jamrozik et al., 2017, 2021). This adjustment was implemented to enable selection on CH_4_ as a standalone selection criterion. However, complete genetic independence may not be achieved in practice because the recursive adjustment relies on estimated parameters using a subset of the population. Consequently, small residual associations between MEF and production traits may remain and could introduce minor indirect production information into MEF GEBV predictions. Nevertheless, because the estimated genetic correlations were zero after the recursive modeling approach (Jamrozik et al., 2017, 2021; Rojas de Oliveira et al., 2024), any impact on predictive performance is expected to be minimal. Genomic evaluations are reported as relative breeding values (**RBV**), where higher RBV indicate more desirable, lower-emitting animals, and MEF is the only value published from the Canadian genomic evaluation system.

Validation of genomic evaluations is crucial in ensuring the accuracy, reliability, and effectiveness of genetic selection programs. It verifies whether GEBVs reflect true phenotypic performance, helping to minimize bias and support informed breeding decisions. Robust validation also fosters stakeholder confidence, particularly when introducing new traits or transitioning to updated models or different approaches, such as single-step evaluations (Ooi et al., 2024). A variety of methods are available for validating genomic evaluations, including linear regression (Legarra and Reverter, 2018), genetic group sampling (Hickey et al., 2008), the Interbull GEBV test (Mäntysaari et al., 2010; Sullivan, 2023), and Mendelian sampling (Tyrisevä et al., 2018; Sullivan, 2019). Typically, both full and truncated datasets are created, where the latter omits recent years of phenotypic data (e.g., 1 to 4 years) to enable forward prediction. An additional validation approach involves assessing whether genetic categories defined by breeding values correspond with actual trait performance in an independent validation population (McNeel et al., 2017; Stachowicz et al., 2024). Therefore, this study aimed to validate MEF evaluations using RBV for MPR and MEF obtained from the April 2023 official genomic evaluation (the first official publication of MEF) in genotyped cows based on: (1) CH_4MIR_ (g/d) included in the December 2024 genomic evaluation, and (2) CH_4_ production recorded via the GreenFeed system (**CH_4GF_**; g/d).

Within the December 2024 genomic evaluation of MPR and MEF there were 687,069 primiparous Holstein cows with 998,573 CH_4MIR_ records, of which 96,235 were genotyped. Cows for the validation dataset were determined by identifying cows with CH_4MIR_ (g/d) phenotypic records included in the December 2024 genomic evaluation, but not in the April 2023 genomic evaluation. The resulting dataset consisted of 147,928 primiparous Holstein cows with 204,990 CH_4MIR_ records. Phenotypes of CH_4MIR_ are routinely predicted for first lactation Holstein cows between 120 and 185 DIM as described by Rojas de Oliveira et al. (2024). To account for known environmental effects, CH_4MIR_ phenotypes were corrected for the fixed effect of herd-year-season of calving, and a minimum of 5 cows per herd-year-season level were required. After applying this criterion, 112,384 cows with 161,001 records remained. For cows with multiple CH_4MIR_ records available, an average was calculated. Next, respective RBV of MPR and MEF from the April 2023 genomic evaluations were merged with the cows' phenotypic data. This step resulted in 111,396 cows (born between 2020 and 2022), of which 21,629 were genotyped. Given that these cows did not have phenotypes available during the April 2023 genomic evaluation, the RBV were calculated based on the cow's genomic parent average. Genomic evaluations of MPR and MEF are expressed as RBV, standardized to a mean of 100 with an SD of 5, where higher values are defined as more desirable across all traits. Accordingly, the scale for MPR and MEF is reversed so that a higher RBV reflects a more favorable cow with lower CH_4MIR_. For the validation analysis, only genotyped cows with reliabilities ≥70% for both MPR and MEF RBV were retained. The final validation dataset comprised 20,913 cows with CH_4MIR_ phenotypes recorded since April 2023, and RBV of MPR and MEF from the April 2023 genomic evaluation results.

For the analysis with CH_4MIR_ phenotypic records, validation cows (n = 20,913) were categorized into quintile groups (**Q1–Q5**) for both MPR and MEF based on RBV: Q1 (<20th percentile), Q2 (20th–40th percentile), Q3 (40th–60th percentile), Q4 (60th–80th percentile), and Q5 (>80th percentile). Quintile-based grouping ensured approximately balanced sample sizes across categories and allowed evaluation of trends across the RBV distribution. Descriptive statistics of the quintile groups of MPR and MEF are presented in Table 1. To identify suitable statistical methods for comparing quintile means, normality and homoscedasticity were assessed, and residual independence was assumed. Model assumptions were evaluated using residuals. Residual distributions for CH_4MIR_ were approximately symmetric for both MPR and MEF models, with skewness values close to zero (MPR: −0.06; MEF: −0.08). Excess kurtosis values were moderately elevated (MPR: 1.41; MEF: 1.44), indicating mild tail heaviness but remaining within accepted thresholds for approximate normality in large samples. Homoscedasticity was assessed by examining model residuals within each RBV quintile group, where residual standard deviations, variances, and coefficients of variation were calculated and compared across quintiles. Residual variability was highly consistent for both traits, with small maximum-to-minimum variance ratios (MPR: 1.17; MEF: 1.08) and SD ratios (MPR: 1.08; MEF: 1.04). Coefficients of variation were also comparable across quintiles (MPR: 0.054–0.062; MEF: 0.057–0.060), indicating no substantial heteroscedasticity across quintiles. As all assumptions were met, quintile comparisons were performed using one-way ANOVA based on the following linear model:

*y_ij_* = *μ* + *G_i_* + *ε_ij_*,

where *y_i_* is the adjusted methane phenotype of cow *j*, *μ* is the overall mean of the adjusted methane phenotype, *G_i_* is the fixed effect of the RBV group, and *ε_ij_* is the residual error. This model was used for all ANOVA analyses described in this study.

**Table 1:** Mean ± SD of adjusted MIR-predicted methane (CH_4MIR_) and number of cows across quintiles based on RBV of methane production and methane efficiency (n = 20,913)

Quintile^1^	Cows	Methane production (g/d)		Methane efficiency (g/d)	
		Mean	SD	Mean	SD
Q1	4,183	513.07	27.60	510.51	28.14
Q2	4,183	503.95	28.26	503.75	28.82
Q3	4,183	498.24	27.67	497.47	29.15
Q4	4,182	491.66	28.28	492.55	29.26
Q5	4,182	481.72	29.80	484.36	29.12

1 Q1 (<20th percentile), Q2 (20th–40th percentile), Q3 (40th–60th percentile), Q4 (60th–80th percentile), and Q5 (>80th percentile).

To assess differences among quintiles, pairwise comparisons were performed using estimated marginal means implemented with the emmeans package in R (version 4.1.3, R Core Team, Vienna, Austria; Lenth and Piaskowski, 2025), with Tukey adjustment applied to account for multiple comparisons among all quintiles. Significant statistical differences were considered at α < 0.05.

In addition to the quintile-based analysis, genetic categories defined by RBV thresholds were evaluated to directly compare animals with the highest RBV against other groups. Animals were categorized into 5 RBV-based groups: low (RBV <90; n = 353), low–medium (90 ≤ RBV <95; n = 3,465), medium (95 ≤ RBV ≤105; n = 16,032), medium–high (105 < RBV ≤110; n = 1,011), and high (RBV >110; n = 52). Diagnostic assessments of model assumptions showed similar patterns to those observed for the quintile-based groupings. As these RBV-based categories resulted in unbalanced group sizes, differences among categories were tested by planned contrasts to evaluate targeted comparisons between the highest RBV group and each of the remaining categories using estimated marginal means and Dunnett's test implemented with the emmeans package in R (Lenth and Piaskowski, 2025). Significant statistical differences were considered at α < 0.05.

Furthermore, to estimate the expected reduction in CH_4MIR_ per unit increase in RBV, cows were grouped by individual RBV values (e.g., 85, 86). Only RBV classes with a minimum of 20 cows were retained to avoid instability due to small-sized groups. For each retained RBV class, the mean adjusted CH_4MIR_ was calculated. These class means were then regressed on RBV using weighted linear regression, with weights equal to the number of cows within each RBV class:

$${\bar{y}}_{i}=\beta_{0\;}+\beta_{1\;}RBV_{i\;}+\varepsilon_{i\;},$$

where ${\bar{y}}_{i}$ represents the mean CH_4MIR_ for RBV class *i*, *β*_0_ is the intercept, *β*_1_ is the regression coefficient describing the expected change in mean CH_4MIR_ per 1-unit increase in RBV, and *RBV_i_* is the corresponding RBV of class *i*.

Among the 20,913 validation cows included in the above analysis, 134 first lactation cows from the Ontario Dairy Research Centre (Elora, ON, Canada) with weekly CH_4GF_ (g/d) records were identified (120–185 DIM). These cows were part of the validation population because their phenotypes were not included in the April 2023 genomic evaluation but became available in the December 2024 dataset. Similar to CH_4MIR_ phenotypes, the daily CH_4GF_ records were adjusted for the fixed effect of year-season of calving. These adjusted daily values were then averaged to obtain weekly means per cow. Due to the smaller sample size, RBV of both MPR and MEF were divided into tertiles: T1 (<33rd percentile; n = 45), T2 (33rd–66th percentile; n = 45), and T3 (>66th percentile; n = 44). Tertile-based grouping ensured approximately balanced sample sizes across categories and allowed evaluation of trends across the RBV distribution. Once again, normality and homoscedasticity were evaluated using residuals, whereas residual independence was assumed. Across RBV MPR and MEF tertiles, skewness (MPR: −0.18; MEF: −0.20) and excess kurtosis (MPR: −0.45; MEF: −0.56) values fell within accepted thresholds for approximate normality. No substantial heteroscedasticity was observed across tertiles, with small maximum-to-minimum variance ratios (MPR: 1.06; MEF: 1.21) and SD ratios (MPR: 1.03; MEF: 1.10), and comparable coefficients of variation across tertiles (MPR: 0.131–0.139; MEF: 0.133–0.139). As all assumptions were met, tertile comparisons were performed using one-way ANOVA. Pairwise comparisons were again applied to assess differences among tertiles using estimated marginal means implemented with the emmeans package in R (Lenth and Piaskowski, 2025), with Tukey adjustment applied. Given the smaller sample size, a significance threshold of α < 0.10 was used.

Genetic categories defined by RBV thresholds were also evaluated to directly compare animals with the highest RBV against other groups. Animals were categorized into 3 RBV-based groups for the CH_4GF_ data: low (RBV <95; n = 21 for MPR; n = 11 for MEF), medium (95 ≤ RBV ≤ 105; n = 102 for MPR; n = 108 for MEF), and high (RBV >105; n = 11 for MPR; n = 15 for MEF). The same RBV-based analytical approach described for the CH_4MIR_ dataset was applied, including planned contrasts to compare the highest RBV group against the remaining categories using estimated marginal means and Dunnett's test implemented with the emmeans package in R (Lenth and Piaskowski, 2025). The same significance threshold used for the tertile-based analysis (α < 0.10) was applied.

The mean ± SD CH_4MIR_ for the 20,913 validation cows (120–185 DIM) was 497.73 ± 30.27 g/d. The mean reliability ± SD of MPR and MEF RBV was 72.42% ± 1.61% for both traits, ranging from 70% to 86%. These values are consistent with the range of CH_4MIR_ means reported previously (Dehareng et al., 2012; Fresco et al., 2024; McParland et al., 2024; Rojas de Oliveira et al., 2024). Quintile group had a highly significant effect on CH_4MIR_ for both MPR and MEF (one-way ANOVA, *P* < 2.2 × 10^−16^; Figure 1).

**Figure 1:**
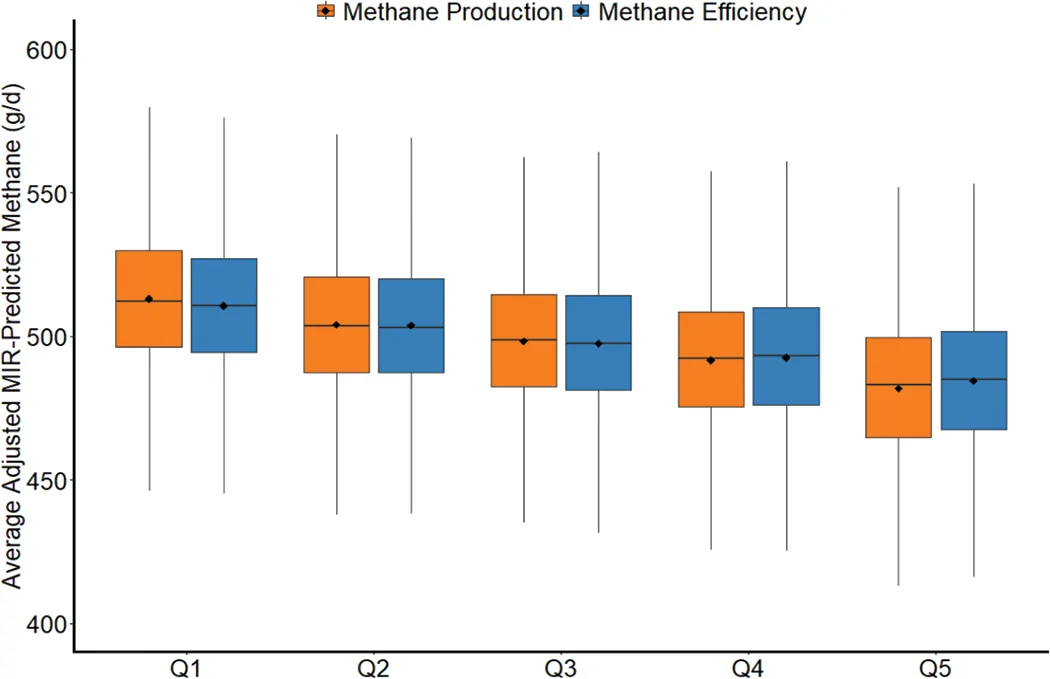
Boxplot of average adjusted MIR-predicted methane (g/d) across relative breeding value quintiles for methane production and methane efficiency in validation cows (n = 20,913). Quintiles were defined as Q1 (<20th percentile), Q2 (20th–40th percentile), Q3 (40th–60th percentile), Q4 (60th–80th percentile), and Q5 (>80th percentile). Pairwise comparisons among quintiles were significant for both traits (*P* < 0.05). Top and bottom limits of the box represent the interquartile range (25th to 75th percentile), the black lines inside each boxplot represent the median value, and the black diamonds inside each boxplot represent the mean value. Whiskers are the minimum and maximum values within 1.5 × interquartile range.

For MPR, mean CH_4MIR_ decreased progressively across RBV quintiles, from 513.1 g/d in Q1 to 481.7 g/d in Q5 (Figure 1). This represents a reduction of 31.4 g/d (6.1%) between the lowest and highest quintile. A similar pattern was observed for MEF. Mean CH_4MIR_ decreased from 510.5 g/d in Q1 to 484.4 g/d in Q5, corresponding to a reduction of 26.1 g/d (5.1%) across the RBV distribution. For both MPR and MEF, all pairwise comparisons among quintiles were statistically significant (Tukey-adjusted *P* < 0.001). Overall, these results demonstrate a consistent decline in methane emissions as RBV for both MPR and MEF increase.

Planned contrasts based on RBV thresholds compared each genetic category against the high group for both MPR and MEF. In both traits, cows in the high category showed the lowest CH_4MIR_, whereas progressively higher emissions were observed as RBV decreased. All contrasts were statistically significant (*P* < 0.005), confirming that the genomic evaluations for MPR and MEF effectively discriminate cows based on methane emission potential.

Linear regression analysis further supported these findings, showing favorable negative slopes for both MPR (−2.6 g/d; R^2^ = 0.99) and MEF (−2.1 g/d; R^2^ = 0.99), with regression predicting an average CH_4MIR_ reduction of 10.5 g/d for every 5-point increase in MEF RBV. This aligns with Rojas de Oliveira et al. (2024), who reported a reduction of 7.55 g/d in daughters per 5-point increase in sire MEF RBV, equivalent to ∼15 g/d when selection is based on female RBV. These results further highlight the potential of genomic selection to reduce CH_4_ emissions in Canadian Holsteins.

For the 134 cows with CH_4GF_ records, the mean ± SD CH_4GF_ and CH_4MIR_ were 425.66 ± 59.01 and 496.98 ± 21.26 g/d, respectively, whereas RBV reliability averaged 72.88% ± 1.30% (range: 70%–76%). One-way ANOVA indicated significant differences in CH_4GF_ among tertiles for both MPR (*P* = 0.02) and MEF (*P* = 0.06; Figure 2), applying a significance threshold of *P* < 0.10. Mean CH_4GF_ decreased across RBV tertiles for both traits, declining from 445 g/d in Q1 to 413 g/d in Q3 for MPR and from 442 g/d in Q1 to 415 g/d in Q3 for MEF. Pairwise comparisons indicated significant differences between Q1 and Q2 (*P* = 0.08) and between Q1 and Q3 (*P* = 0.03) for MPR. For MEF, a significant difference was observed between Q1 and Q3 (*P* = 0.07), whereas other contrasts were not significant. Overall, the direction and magnitude of differences were consistent with the expected inverse relationship between RBV and methane emissions.

**Figure 2:**
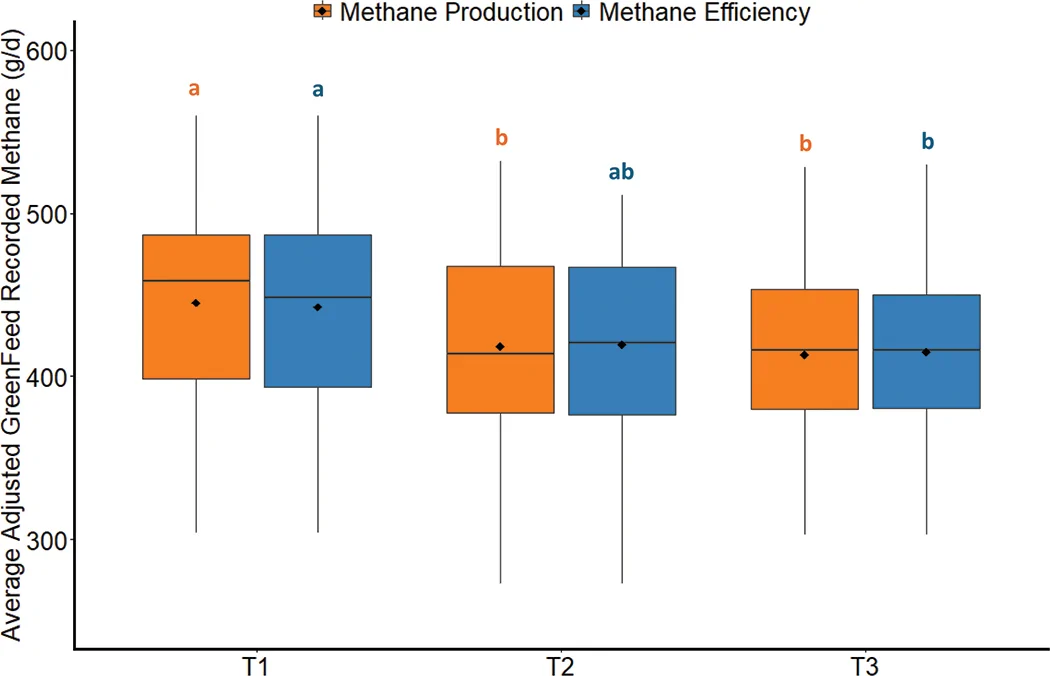
Boxplot of average adjusted GreenFeed-recorded methane emissions (g/d) across relative breeding value tertiles for methane production and methane efficiency in validation cows (n = 134) from the Ontario Dairy Research Centre (Elora, ON, Canada). Tertiles were defined as T1 (<33rd percentile), T2 (33rd–66th percentile), and T3 (>66th percentile). Different lowercase letters indicate significant differences (*P* < 0.10). Top and bottom limits of the box represent the interquartile range (25th to 75th percentile), the black lines inside each boxplot represent the median value, and the black diamonds inside each boxplot represent the mean value. Whiskers are the minimum and maximum values within 1.5 × interquartile range.

Planned contrasts based on RBV threshold categories indicated lower CH_4GF_ emissions in the high RBV group compared with the low group for both MPR (*P* = 0.03) and MEF (*P* = 0.09), whereas differences between the high and medium were only significant for MEF (*P* = 0.10). The difference between low and high categories was 52.2 g/d (11%) for MPR and 46.3 g/d (10%) for MEF, which is comparable to the reductions observed for CH_4MIR_.

The relationship between CH_4MIR_ and CH_4GF_ was also explored, with previous studies estimating a phenotypic correlation of 0.70 (Rojas de Oliveira et al., 2024) and a genetic correlation of 0.92 (Van Doormaal et al., 2023). Relative differences between CH_4MIR_ and CH_4GF_ were consistently below 20% across groups, with CH_4MIR_ tending to slightly overestimate CH_4GF_. A smaller phenotypic standard deviation was observed for CH_4MIR_ compared with CH_4GF_ suggesting a compression of phenotypic variability. If additive genetic variance is similarly reduced, the genetic standard deviation and thus the theoretical response to selection would also decrease. Although this may affect the magnitude of expected genetic gain, the validity of selection depends primarily on accurate ranking rather than absolute scale. Differences between measures may also reflect discrepancies in timing and number of records. The CH_4MIR_ was derived from multiple test-day records collected across 120 to 185 DIM, resulting in repeated measurements spanning this period, whereas CH_4GF_ was recorded over a single consecutive week. Nevertheless, CH_4MIR_ proved to be a reliable and scalable proxy for CH_4_ emissions, particularly suitable for large-scale on-farm data collection, though expanded data would enhance the precision of these estimates.

Although the results in this study strongly support the effectiveness of MEF evaluations, it should be noted that the dataset was not entirely independent of the April 2023 national evaluation. Records from relatives of validation cows may have contributed indirectly through pedigree and genomic information. This limitation is inherent to forward prediction validation, where phenotypes are excluded from the truncated dataset but pedigree and genomic structure remain intact. Similar biases were observed by McNeel et al. (2017) and Stachowicz et al. (2024). Future validation using external, fully independent datasets would further strengthen the robustness of the findings.

Overall, the findings of this study provide strong evidence that genomic evaluation of MEF using CH_4MIR_ effectively differentiates cows based on their CH_4_ emissions. The observed decreasing trend in CH_4MIR_ and CH_4GF_ with increasing RBV of MPR and MEF and statistical differences between genetic categories of MEF highlight the potential of genetic selection for reducing CH_4_ emissions in dairy cattle. Furthermore, selecting cows with higher RBV for MEF offers a promising long-term and cost-effective approach to CH_4_ mitigation. Future research should focus on increasing validation sample sizes and incorporating external datasets to refine these genomic selection strategies further.
